# A Plant-Based High-Carbohydrate, Low-Fat Diet in Overweight Individuals in a 16-Week Randomized Clinical Trial: The Role of Carbohydrates

**DOI:** 10.3390/nu10091302

**Published:** 2018-09-14

**Authors:** Hana Kahleova, Sara Dort, Richard Holubkov, Neal D. Barnard

**Affiliations:** 1 Physicians Committee for Responsible Medicine, 5100 Wisconsin Ave, N.W. Ste.400, Washington, DC 20016, USA; sdort@pcrm.org (S.D.); nbarnard@pcrm.org (N.D.B.); 2 School of Medicine, University of Utah, Salt Lake City, UT 84132, USA; richard.holubkov@hsc.utah.edu; 3 Adjunct Faculty, George Washington University School of Medicine and Health Sciences, Washington, DC 20016, USA

**Keywords:** carbohydrates, diet, fiber, nutrition, plant-based, vegan

## Abstract

The effects of carbohydrates on body weight and insulin sensitivity are controversial. In this 16-week randomized clinical trial, we tested the role of a low-fat, plant-based diet on body weight, body composition and insulin resistance. As a part of this trial, we investigated the role of changes in carbohydrate intake on body composition and insulin resistance. Participants (*n* = 75) were randomized to follow a plant-based high-carbohydrate, low-fat (vegan) diet (*n* = 38) or to maintain their current diet (*n* = 37). Dual-energy X-ray absorptiometry was used to measure body composition. Insulin resistance was assessed with the Homeostasis Model Assessment (HOMA-IR) index. A repeated measure ANOVA model was used to test the between-group differences from baseline to 16 weeks. A linear regression model was used to test the relationship between carbohydrate intake, and body composition and insulin resistance. Weight decreased significantly in the vegan group (treatment effect −6.5 [95% CI −8.9 to −4.1] kg; Gxt, *p* < 0.001). Fat mass was reduced in the vegan group (treatment effect −4.3 [95% CI −5.4 to −3.2] kg; Gxt, *p* < 0.001). HOMA-IR was reduced significantly in the vegan group (treatment effect −1.0 [95% CI −1.2 to −0.8]; Gxt, *p* = 0.004). Changes in consumption of carbohydrate, as a percentage of energy, correlated negatively with changes in BMI (*r* = −0.53, *p* < 0.001), fat mass (*r* = −0.55, *p* < 0.001), volume of visceral fat (*r* = −0.35, *p* = 0.006), and HOMA (*r* = −0.27, *p* = 0.04). These associations remained significant after adjustment for energy intake. Changes in consumption of total and insoluble fiber correlated negatively with changes in BMI (*r* = −0.43, *p* < 0.001; and *r* = −0.46, *p* < 0.001, respectively), fat mass (*r* = −0.42, *p* < 0.001; and *r* = −0.46, *p* < 0.001, respectively), and volume of visceral fat (*r* = −0.29, *p* = 0.03; and *r* = −0.32, *p* = 0.01, respectively). The associations between total and insoluble fiber and changes in BMI and fat mass remained significant even after adjustment for energy intake. Increased carbohydrate and fiber intake, as part of a plant-based high-carbohydrate, low-fat diet, are associated with beneficial effects on weight, body composition, and insulin resistance.

## 1. Introduction

The prevalence of obesity has reached epidemic proportions. The World Health Organization estimates that more than 1.9 billion adults worldwide have excess body weight [1,2]. As a result, the prevalence of obesity-related diseases is rapidly increasing, and increased body weight is associated with a higher all-cause mortality [3]. Practical and sustainable weight loss strategies are needed.

Poor nutrition habits are a leading contributor to obesity, chronic disease, and premature death in the United States and worldwide [2,4]. It has been estimated that dietary factors, such as high intakes of processed meat products and sodium and low intakes of fruits and vegetables, are associated with roughly half of cardio-metabolic deaths in the United States [5]. In contrast, plant-based diets represent an effective strategy for improving nutrient intake [6], and are associated with decreased all-cause mortality and decreased risk of obesity, type 2 diabetes, and coronary heart disease [7]. 

A vegan diet is characterized by elimination of animal products and is based on the consumption of grains, legumes, vegetables, and fruits. A well-balanced vegan diet can meet all macro and micronutrient intake recommendations and is high in fiber and carbohydrates [8]. Whole grains [9,10], legumes [11], fruits, and vegetables [12,13,14] have shown independent advantages for weight-related outcomes. Systematic reviews and meta-analyses of randomized clinical trials of dietary patterns that are high in carbohydrates but low in glycemic index [15,16], and high in fiber [17] have shown beneficial effects on body weight loss and weight management in overweight people. 

The role of carbohydrates in weight management is controversial. Observational studies suggest that high-carbohydrate diets are associated with healthy body weight, within the normal BMI range of 18.5 to 24.9, while replacing carbohydrates with fat can cause weight gain. In this 16-week randomized controlled trial, we explored the effects of changes in carbohydrate and fiber intake, as part of a plant-based, high-carbohydrate, low-fat diet, on weight control, body composition, and insulin resistance in overweight individuals. Our hypothesis was that high carbohydrate and fiber intakes in the context of a plant-based diet will be associated with weight loss, reduction in fat mass, and decrease in insulin resistance.

## 2. Materials and Methods

### 2.1. Study Design

The study was conducted between October 2016 and June 2017, using a single-center, randomized, open parallel design. The study protocol was approved by the Ethics Committee of the Chesapeake Institutional Review Board on 12 October 2016. The protocol identification number is Pro00018983. All participants gave written informed consent. Registration on ClinicalTrials.gov was initiated on 20 October 2016 (Identifier: NCT02939638).

Participants were adults with a BMI between 28 and 40 kg/m^2^. They were recruited through local newspaper advertisements, radio advertisements, healthcare professional referrals, mailing lists, and flyers. Comorbidities or recent use of medications that alter appetite or body weight precluded participation, as did pregnancy, recent smoking or recreational drug use, evidence of an eating disorder, alcohol consumption above two drinks a day, or unwillingness to comply with study requirements. 

### 2.2. Randomization and Study Groups

A computer-generated randomization protocol assigned participants randomly in a 1:1 ratio to an intervention group or a control group. The randomization protocol could not be accessed beforehand. Because assignment was done simultaneously, allocation concealment was unnecessary. The participants were not blinded to their group assignment. No meals were provided. The intervention group was asked to follow a low-fat vegan diet consisting of vegetables, grains, legumes, and fruits. Each participant met for 1-h with a registered dietitian to establish an appropriate diet plan. Thereafter, participants attended weekly 1-h meetings for nutrition and cooking instruction conducted by a physician and a registered dietitian and/or a cooking instructor. Participants were instructed to avoid animal products and added oils. Prescribed daily fat intake was 20–30 g. There were no limits on energy or carbohydrate intake. Participants prescribed the plant-based dietary pattern were invited to weekly classes. The control group participants were asked to maintain their current diets, which included meat and dairy products, for the 16-week intervention period. All study participants were asked not to alter their physical activity and to continue their preexisting medication regimens for the duration of the study, except as modified by their personal physicians.

### 2.3. Measurements 

All measurements were performed at baseline and 16 weeks after an overnight fast. Height and weight were measured using a stadiometer and a periodically calibrated scale accurate to 0.1 kg, respectively. Body composition was assessed using a DXA scan (iDXA; GE Healthcare, Chicago, IL, USA). Insulin resistance was calculated as HOMA-IR (Homeostasis Model Assessment) index [18]. Physical activity was assessed by the International Physical Activity Questionnaire (IPAQ) [19]. Dietary intake was measured using a three-day dietary record at baseline and 16 weeks. Dietary data were collected and analyzed by a registered dietician, using Nutrition Data System for Research version 2016, developed by the Nutrition Coordinating Center (NCC), University of Minnesota, Minneapolis, MN [20]. Laboratory measurements and statistical analyses were made by staff members blind to group assignment.

### 2.4. Statistical Analysis

The intention-to-treat analysis included all participants. A repeated measure ANOVA model was used to test the between-group differences from baseline to 16 weeks. Factors group, subject, and time were included in the model. Interaction between group and time (Gxt) was calculated for each variable. Regression analyses were calculated for the relationship between changes in reported carbohydrate intake on one side, and body mass index, body composition and insulin resistance on the other, first unadjusted, and then adjusted for changes in energy intake in both groups combined. The intake of carbohydrates and markers of carbohydrate quality were used as predictors of changes in body mass index, body composition, and insulin resistance.

## 3. Results

### 3.1. Characteristics of the Participants

**Figure 1:**
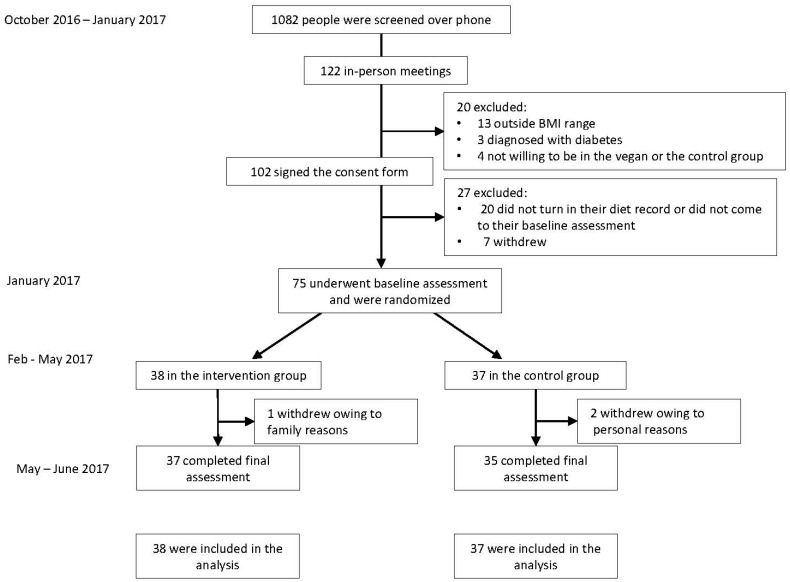
Enrollment of the Participants and Completion of the Study.

The flow of participants through the study is shown in Figure 1. The mean age was 53.2 ± 12.6 years. The majority of our study participants (89%, *n* = 67) were women. Baseline characteristics of the study population are shown in Table 1. Of 75 participants who were randomized, 96% (*n* = 72) completed the entire study. Two participants dropped out from the control group owing to personal reasons and one participant dropped out of the intervention group due to a family emergency.

### 3.2. Dietary Intake and Physical Activity

Dietary intake and physical activity are shown in Table 2. Physical activity did not change substantially in either group. Both groups reduced reported energy intake, with no significant difference between groups (*p* = 0.69). Total carbohydrate intake did not change in the control group, while it increased significantly in the vegan group, both as absolute intake and as a percent of total energy (treatment effect +70.6; 95% CI +28.8 to +115 g/day; *p* = 0.001). Total and insoluble fiber increased significantly in the vegan group (treatment effect +15.3; 95% CI +8.0 to +22.6 g/day; *p* < 0.001, and +12.5; 95% CI +7.16 to +17.7 g/day; *p* < 0.001, respectively). Glycemic index decreased similarly in both groups. Consumption of starch decreased in the control group and increased in the vegan group (treatment effect +49.6; 95% CI +24.9 to +74.2 g/day; *p* < 0.001). Sucrose intake did not change significantly in either group. Glucose and fructose consumption increased, while lactose intake decreased significantly in the vegan group.

### 3.3. Body Composition and Insulin Resistance

**Figure 2:**
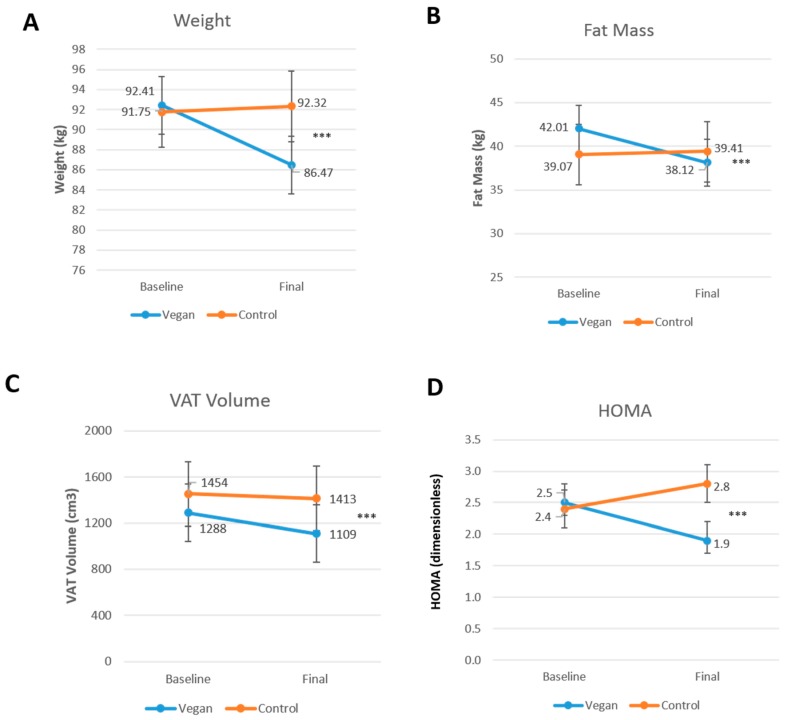
Changes in body weight, fat mass, and insulin resistance in the vegan and control group at baseline and after 16 weeks. (**A**) Body Weight, Gxt *p* < 0.001; (**B**) Fat Mass, Gxt *p* < 0.001; (**C**) Visceral Adipose Tissue (VAT) Volume, Gxt *p* < 0.001; and (**D**) Homeostatic Model Assessment Insulin Resistance (HOMA-IR), Gxt *p* = 0.004. Gxt is interaction between group and time from the ANOVA model. *** for *p* < 0.001. Data are given as means with 95% confidence intervals.

Body mass index and body weight decreased significantly only in the vegan group (treatment effect −2.0; 95% CI −2.6 to −1.5 kg/m^2^; Gxt, *p* < 0.001; Figure 2A; and, treatment effect −6.5; 95% CI −8.9 to −4.1 kg; Gxt, *p* < 0.001, respectively). Fat mass and visceral fat volume, were reduced only in the vegan group (treatment effect −4.3; 95% CI −5.4 to −3.2 kg; Gxt, *p* < 0.001; Figure 2B; and treatment effect −224; 95% CI −328 to −120 cm^3^; Gxt, *p* < 0.001; Figure 2C, respectively). HOMA-IR was reduced significantly only in the vegan group (treatment effect −1.0; 95% CI −1.2 to −0.8; Gxt, *p* = 0.004; Figure 2D). 

### 3.4. Association between Carbohydrate Quantity and Body Composition and Insulin Resistance

**Figure 3:**
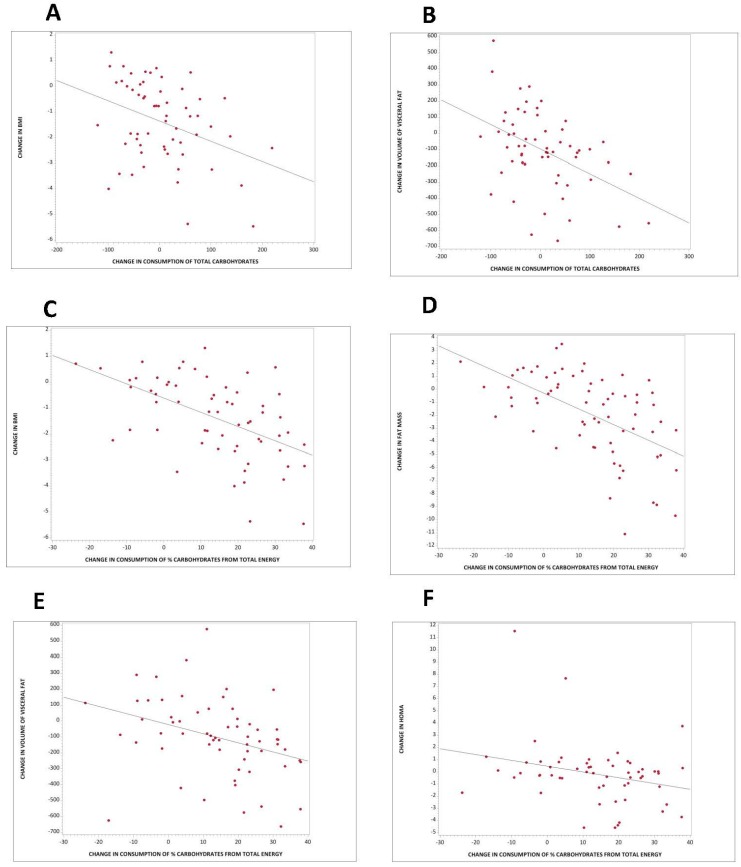

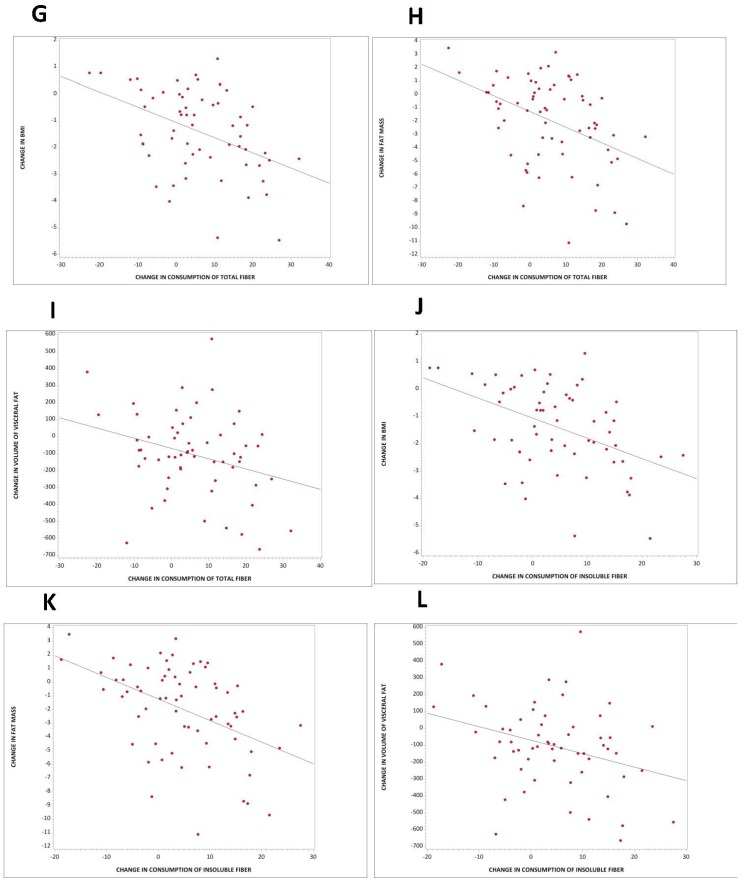
Regression models for changes in carbohydrate intake and changes in body composition, fat mass, and insulin resistance in both groups combined. (**A**) Total carbohydrate intake and change in BMI: *r* = −0.36; *p* = 0.005; (**B**) Total carbohydrate intake and change in volume of visceral fat: *r* = −0.45; *p* < 0.001; (**C**) % carbohydrate intake from total energy and change in BMI: *r* = −0.53, *p* < 0.001; (**D**) % carbohydrate intake from total energy and change in fat mass: *r* = −0.55, *p* < 0.001; (**E**) % carbohydrate intake from total energy and change in volume of visceral fat: *r* = −0.35, *p* = 0.006; (**F**) % carbohydrate intake from total energy and change in HOMA: *r* = −0.27, *p* = 0.04; (**G**) Total fiber intake and change in BMI: *r* = −0.43, *p* < 0.001; (**H**) Total fiber intake and change in fat mass: *r* = −0.42; *p* < 0.001; (**I**) Total fiber intake and change in volume of visceral fat: *r* = −0.29; *p* = 0.003; (**J**) Intake of insoluble fiber and change in BMI: *r* = −0.46, *p* < 0.001; (**K**) Intake of insoluble fiber and change in fat mass: *r* = −0.46, *p* < 0.001; (**L**) Intake of insoluble fiber and change in volume of visceral fat: *r* = −0.32, *p* = 0.01.

Changes in consumption of total carbohydrates as absolute quantity correlated negatively with changes in BMI and volume of visceral fat (*r* = −0.36, *p* = 0.005, Figure 3A; and *r* = −0.45, *p* < 0.001, Figure 3B, respectively). For every 100 g of additional carbohydrates consumed per day, there was a decrease in BMI by 0.40 (95% CI −0.78 to −0.001) kg/m^2^, and a decrease in volume of visceral fat by 91 (95% CI −149 to −33) cm^3^. These associations remained significant even after adjustment for energy intake (*r* = −0.43, *p* < 0.001; and *r* = −0.36, *p* = 0.005, respectively). The correlation between changes in consumption of total carbohydrates and changes in visceral fat remained significant even after adjustment for changes in BMI (*r* = −0.31, *p* = 0.02).

Changes in consumption of carbohydrate as a percent of total energy correlated negatively with changes in BMI (*r* = −0.53, *p* < 0.001), fat mass (*r* = −0.55, *p* < 0.001), volume of visceral fat (*r* = −0.35, *p* = 0.006), and HOMA (*r* = −0.27, *p* = 0.04). For every 10% more carbohydrates consumed per day from total energy, there was a decrease in BMI of 0.55 (95% CI −0.78 to −0.32) kg/m^2^ (Figure 3C), a reduction in fat mass of 1.21 (95% CI −1.66 to −0.80) kg (Figure 3D), a decrease in volume of visceral fat of 57 (95% CI −97 to −17) cm^3^ (Figure 3E), and a reduction in HOMA of 0.48 (95% CI −0.92 to −0.03; Figure 3F). These associations remained significant even after adjustment for energy intake (*r* = −0.53, *p* < 0.001 for BMI; *r* = −0.55, *p* < 0.001 for fat mass; *r* = −0.40, *p* = 0.002 for visceral fat; and *r* = −0.27, *p* = 0.04 for HOMA).

### 3.5. Association between Carbohydrate Quality and Body Composition and Insulin Resistance

Changes in consumption of total, and particularly insoluble, fiber correlated negatively with changes in BMI (*r* = −0.43, *p* < 0.001; and *r* = −0.46, *p* < 0.001, respectively), fat mass (*r* = −0.42, *p* < 0.001; and *r* = −0.46, *p* < 0.001, respectively), and volume of visceral fat (*r* = −0.29, *p* = 0.03; and *r* = −0.32, *p* = 0.01, respectively). Every additional 10 g of total fiber per day was associated with a decrease in BMI of 0.24 (95% CI −0.45 to −0.03) kg/m^2^ (Figure 3G), a reduction in fat mass of 0.54 (95% CI −0.97 to −0.11) kg (Figure 3H), and a reduction in volume of visceral fat of 34.8 (95% CI −68.2 to −14.0) cm^3^ (Figure 3I). Every 10 g of additional insoluble fiber per day was associated with a decrease in BMI of 0.41 (95% CI −0.69 to −0.13) kg/m^2^ (Figure 3J), a reduction in fat mass of 0.92 (95% CI −1.50 to −0.35) kg (Figure 3K), and a reduction in volume of visceral fat of 55.7 (95% CI −101 to −10.5) cm^3^ (Figure 3L). The association between changes in total and insoluble fiber and changes in BMI (*r* = −0.43, *p* < 0.001; and *r* = −0.46, *p* < 0.001, respectively) and fat mass (*r* = −0.45, *p* < 0.001; and *r* = −0.49, *p* < 0.001, respectively) remained significant even after adjustment for energy intake. After adjustment for changes in BMI, these associations were no longer significant.

Changes in consumption of starch correlated negatively with changes in BMI (*r* = −0.25, *p* = 0.05), and volume of visceral fat (*r* = 0.42, *p* < 0.001). These associations remained significant even after adjustment for energy intake (*r* = −0.30, *p* = 0.02; and *r* = −0.34, *p* = 0.009, respectively). The latter remained significant even after adjustment for changes in BMI (*r* = −0.35, *p* = 0.006).

Changes in consumption of lactose correlated positively with changes in BMI (*r* = 0.30, *p* = 0.02), fat mass (*r* = 0.27, *p* = 0.03), and HOMA (*r* = 0.25, *p* = 0.05). The former two associations remained significant even after adjustment for energy intake (*r* = 0.31, *p* = 0.02; and *r* = 0.26, *p* = 0.03, respectively).

## 4. Discussion

### 4.1. Main Findings

This 16-week randomized controlled study demonstrated that increased consumption of carbohydrates and dietary fiber, as part of a plant-based high-carbohydrate, low-fat diet, was associated with reduced body weight, fat mass, and insulin resistance in overweight individuals. Changes in consumption of total carbohydrate correlated negatively with changes in BMI and volume of visceral fat, even after adjustment for energy intake. Changes in consumption of percent carbohydrate from total energy correlated negatively with changes in BMI, fat mass, volume of visceral fat, and HOMA, even after adjustment for energy intake. The association between changes in consumption of total carbohydrate and changes in visceral fat remained significant even after adjustment for changes in BMI, while the association with changes in HOMA was mainly driven by changes in BMI.

Changes in consumption of total and insoluble fiber were negatively associated with changes in BMI, fat mass, and volume of visceral fat. The associations between total and insoluble fiber with BMI and fat mass remained significant even after adjustment for energy intake. Additionally, changes in consumption of starch correlated negatively with changes in BMI, and volume of visceral fat, even after adjustment for energy intake. Interestingly, changes in consumption of lactose correlated positively with changes in BMI, fat mass, and HOMA; the former two remained significant even after adjustment for energy intake.

### 4.2. Carbohydrate Quantity in Weight Regulation, Body Composition, and Insulin Resistance

Our study demonstrated a negative correlation between changes in consumption of both absolute amount and % carbohydrate from total energy and weight changes. These results support previous research on the effect of a plant-based high-carbohydrate diet in weight regulation and body composition [21]. A 2005 14-week randomized control trial (*n* = 64) compared a low-fat vegan diet to a diet that followed National Cholesterol Education Program Guidelines (control), and found a significant decrease in weight loss in the vegan group compared with the control group. Those who followed the low-fat vegan diet (10% of energy from fat, 15% protein, 75% carbohydrates) lost an average of 5.8 kg compared with 3.8 kg for the control group (*p* = 0.012) [22]. Similarly, a 7-day residential dietary intervention program conducted in 2014 found that a low-fat, high-fiber, high-carbohydrate diet (~80% of calories) produced a mean weight loss of 1.4 kg in just 7 days (*p* < 0.001) [23].

Many studies have suggested the use of ad libitum low-fat, high-carbohydrate diets as an effective treatment for weight control and body composition [22,23,24,25,26,27,28]. A 2000 meta-analysis examined 16 intervention trials that compared low-fat diets with control groups that consumed their habitual diets and found low-fat diets, high in fiber-rich carbohydrates, mainly from fruits, vegetables, and whole grains, to be more satiating with fewer calories when compared to foods high in fat. The low-fat intervention groups lost 2.40 kg more in the fixed effects analysis (95% CI; *p* < 0.0001) and 2.52 kg more in the random effects analysis (*p* < 0.0001) when compared to the control groups [24]. Our data showed that increased carbohydrate intake was associated with a decrease in BMI. Similarly, a 2007 review examined carbohydrate quantity and quality in relation to body mass index and concluded that the majority of epidemiologic studies show an inverse association between carbohydrate consumption and BMI [29].

Previous research has demonstrated an important role of dietary carbohydrate quantity in insulin resistance. The Inter99 study, a randomized intervention trial conducted in 2005, examined baseline data from 5675 participants and compared cross-sectional associations between insulin resistance and carbohydrate-related dietary factors. The study found that the intake of total carbohydrate and dietary fiber were inversely associated with HOMA-IR (*p* < 0.05) [30]. Likewise, a 2018 16-week randomized control trial (*n* = 75) compared a low-fat vegan diet (~75% of energy from carbohydrates, 15% protein, and 10% fat) to a control group with no dietary changes, and found that HOMA-IR index fell significantly (*p* < 0.001) in the low-fat vegan group. The changes in HOMA-IR correlated positively with visceral fat volume and changes in BMI (*p* = 0.001 and *p* = 0.009, respectively) [31].

### 4.3. Dietary Fiber in Weight Regulation, Body Composition, and Insulin Resistance

Several meta-analyses have associated consumption of dietary fiber with increased weight loss [32,33]. Our results are in accordance with these studies as we found a negative association between consumption of total, and particularly insoluble fiber, and body weight. In addition, large prospective studies showed a decreased risk of weight gain with a high-fiber diet. Data from the Nurses’ Health Study, a prospective cohort study analyzing data from 74,091 U.S. nurses, showed that over 12 years those with the highest intake of dietary fiber gained an average of 1.52 kg less than those with the lowest intake of dietary fiber. The study showed that increased intake of high-fiber, whole-grain foods was associated with less weight gain [34]. A prospective cohort study followed 27,082 men aged 40–75 years at baseline and showed dietary fiber to be associated with a decreased risk of weight gain, independent of whole-grains. Long-term weight gain was reduced by 5.5 kg for each 20-g/day increment in dietary fiber [35].

Soluble and insoluble fiber both increase satiety, reduce hunger and energy intake, and aid in weight loss [32]. Our study showed that increased intake of insoluble fiber in particular was associated with a decrease in BMI and fat mass. Few studies have investigated the role of insoluble fiber alone; this is because high-fiber foods such as fruit, vegetables, and whole grains have a complex mixture of both soluble and insoluble fibers. A 2007 crossover study (*n* = 31) examined the effect of insoluble fiber on appetite and satiety, and concluded that a high-fiber breakfast cereal containing 33 g of insoluble fiber lead to reduced appetite and increased satiety in healthy men aged 20–35 [36]. 

Our study demonstrated a negative correlation between changes in the consumption of starch and changes in body weight and body composition. These findings support those of investigations into the impact of a high-starch diet on weight regulation. A 1997 study found that high-starch diets had the greatest decrease in body weight and energy intake when compared to high-sucrose or high-fat diets. That study also found that fat mass decreased on the high-starch diet but was unchanged on the high-sucrose and high-fat diets [37]. Similarly, the CARMEN study, a 2000 6-month randomized control trial (*n* = 316), investigated the dietary carbohydrate/fat ratio and the role of simple vs. complex carbohydrates on body weight and blood lipids, and found that participants in the low-fat, high-complex-carbohydrate group lost more weight than those who followed a low-fat, simple-carbohydrate diet or a control diet [38].

Our results showed a positive association between intake of lactose and BMI and HOMA-IR, similar to the results from the Inter99 study, which also showed that the individuals with the higher HOMA-IR values also had a higher BMI, waist circumference, and intake of lactose and protein, but lower intakes of energy, carbohydrates, sucrose, and alcohol [30].

### 4.4. Strengths and Limitations

The design of a randomized control trial enabled us to assess the associations between carbohydrate quantity and quality, as part of a plant-based high-carbohydrate, low-fat diet, and body weight, body composition, and insulin resistance. The study duration was sufficiently long for adaptation to the diet. The low attrition rate suggests the dietary intervention was acceptable and sustainable for the study duration. At the same time, a free-living study represents a challenge for reliable food reporting. The changes in carbohydrate intake were based on 3-day dietary records completed at baseline and again at 16 weeks. These records may have suffered from inaccurate reporting and may not have been fully representative of the diets consumed throughout the intervention. Overweight people frequently under-report their energy intake [39], and this might have been the case particularly in our control group that has not experienced any weight loss in spite of self-reported reduced energy intake. However, we attempted to attenuate any discrepancies by teaching participants how to give detailed reports and by doing random periodic phone calls to evaluate food intake. Finally, this study demonstrates the effectiveness of a plant-based high-carbohydrate, low-fat diet overall, and is not able to prove a causal relationship between carbohydrate intake and metabolic outcomes. In order to prove a causal role of carbohydrates, a specifically-designed randomized clinical trial would need to be done.

## 5. Conclusions

In conclusion, we have demonstrated that increased consumption of carbohydrates and dietary fiber, as part of a plant-based, high-carbohydrate, low-fat diet, were associated with reduced body weight, reduced fat mass, and insulin resistance in overweight individuals. Increased consumption of total carbohydrates was associated with a decrease in BMI and volume of visceral fat, even after adjustment for energy intake. Increased consumption of total and particularly insoluble fiber was associated with a decrease in BMI, fat mass, and volume of visceral fat. Future studies are needed to elucidate the mechanisms behind beneficial metabolic effects of carbohydrates and their role in regulation of body weight, body composition, and insulin resistance.

## Figures and Tables

**Table 1:** Baseline characteristics of study population.

	*n* = 75
**Age (years)**	53.2 ± 12.6
**Sex (number, %)**	
Male	8 (11%)
Female	67 (89%)
**Race (number, %)**	
White	34 (45%)
Black	34 (45%)
Asian, Pacific Islander	4 (5%)
Native American	2 (3%)
N/A—did not disclose	1 (1%)
**Ethnicity (number, %)**	
Non-hispanic	64 (85%)
Hispanic	6 (8%)
N/A—did not disclose	5 (7%)
**Education**	
College	37 (49%)
Graduate degree	37 (49%)
NA	1 (1%)
**Medications**	
Lipid-lowering therapy (%)	9 (12%)
Antihypertensive therapy (%)	18 (24%)
Thyroid medications (%)	9 (12%)

**Table 2:** Changes in dietary intake of carbohydrates during the study. Data are means ± SD. Listed *p*-values are for interactions between group and time assessed by repeated measures ANOVA. * *p* < 0.05, ** *p* < 0.01 and *** *p* < 0.001 for within-group changes from baseline assessed by paired comparison *t*-tests.

Activity and Diet	Control Group		Vegan Group		Treatment Effect	*p*-Value
	Baseline	Week 16	Baseline	Week 16		
Physical activity (METs)	2642 (1476–3809)	2575 (1169–3980)	2207 (1444–2969)	2490 (1586–3395)	+351 (−1143 to +1846)	0.46
Caloric intake (kcal.day^−1^)	1923 (1627–2219)	1582 (1368–1795) **	1851 (1695–2007)	1450 (1249–1652) ***	−60 (−352 to +233)	0.69
Carbohydrates (% of daily energy)	45.5 (42.6–48.4)	46.6 (42.9–50.4)	46.1 (43.5–48.8)	69.6 (67.3–71.8) ***	+22.3 (+17.7 to +26.9)	<0.001
Fats (% of daily energy)	35.6 (32.3–38.9)	35.0 (31.5–38.4)	36.1 (34.0–38.1)	17.5 (15.5–19.4) ***	−17.9 (−22.3 to -13.6)	<0.001
Proteins (% of daily energy)	16.0 (14.94–17.07)	16.99 (15.45–18.52)	16.77 (15.36–18.19)	12.26 (11.26–13.25) ***	−5.50 (−7.90 to -3.11)	<0.001
Cholesterol (mg/day)	290 (220–360)	212 (149–275)	264 (213–315)	6.5 (2.5–10.5) ***	−180 (−278 to −82)	<0.001
Total carbohydrates (g/day)	215 (186–244)	187 (161–212)	217 (198–236)	260 (222–298) *	+70.6 (+28.8 to +115)	0.001
Total fiber (g/day)	25.2 (20.9–29.6)	23.5 (19.6–27.4)	24.2 (21.0–27.4)	37.8 (31.4–44.1) ***	+15.3 (+8.0 to +22.6)	<0.001
Soluble fiber (g/day)	6.42 (5.52–7.31)	6.80 (5.81–7.79)	7.02 (6.09–7.96)	9.87 (7.34–12.4) *	+2.46 (−0.13 to +5.05)	0.06
Insoluble fiber (g/day)	18.5 (14.7–22.3)	16.7 (13.5–19.8)	17.1 (14.6–19.6)	27.7 (23.6–31.7) ***	+12.5 (+7.16 to +17.7)	<0.001
Glycemic index	58.1 (56.2–59.9)	57.4 (55.3–59.5) *	57.7 (55.5–59.9)	54.4 (53.4–55.5) **	−2.6 (−5.7 to +0.5)	0.10
Starch (g/day)	91.6 (78.6–105)	70.8 (59.2–82.4) **	95.6 (82.7–109)	125 (102–147) **	+49.6 (+24.9 to +74.2)	<0.001
Sucrose (g/day)	36.8 (28.6–44.9)	32.9 (25.5–40.3)	36.8 (28.2–45.4)	30.4 (25.9–34.9)	−2.58 (−14.6 to +9.48)	0.67
Glucose (g/day)	18.2 (13.5–22.9)	17.2 (13.1–21.3)	15.7 (13.4–18.0)	20.6 (17.3–23.9) *	+5.86 (+0.53 to +11.2)	0.032
Fructose (g/day)	18.1 (13.0–23.3)	17.5 (13.2–21.8)	17.5 (14.4–20.6)	22.8 (19.4–26.1) *	+5.94 (−0.49 to +12.4)	0.07
Lactose (g/day)	5.99 (3.99–8.00)	6.17 (3.66–8.68)	7.18 (4.93–9.43)	0.56 (0.14–0.98) ***	−6.80 (−10.1 to −3.55)	<0.001
